# Central Neuroplasticity and Decreased Heart Rate Variability after Particulate Matter Exposure in Mice

**DOI:** 10.1289/ehp.0900674

**Published:** 2009-05-20

**Authors:** Hai Pham, Ann C. Bonham, Kent E. Pinkerton, Chao-Yin Chen

**Affiliations:** 1 Department of Pharmacology and; 2 Center for Health and the Environment, University of California at Davis, Davis, California, USA

**Keywords:** air pollution, autonomic function, cardiovascular effect, central neuroplasticity, heart rate variability, particulate matter exposure, transition metal

## Abstract

**Background:**

Epidemiologic studies show that exposure to fine particulate matter [aerodynamic diameter ≤ 2.5 μm (PM_2.5_)] increases the total daily cardiovascular mortality. Impaired cardiac autonomic function, which manifests as reduced heart rate variability (HRV), may be one of the underlying causes. However, the cellular mechanism(s) by which PM_2.5_ exposure induces decreased HRV is not known.

**Objectives:**

We tested the hypothesis that exposure to PM_2.5_ impairs HRV by decreasing the excitability of the cardiac vagal neurons in the nucleus ambiguus. We also detemined the effect of iron on PM-exposure–induced decrease in HRV.

**Methods:**

We measured 24-hr HRV in time domains from electrocardiogram telemetry recordings obtained in conscious, freely moving mice after 3 days of exposure to PM_2.5_ in the form of soot only or iron-soot. In parallel studies, we determined the intrinsic properties of identified cardiac vagal neurons, retrogradely labeled with a fluorescent dye applied to the sinoatrial node.

**Results:**

Soot-only exposure decreased short-term HRV (root mean square of successive difference). With the addition of iron, all HRV parameters were significantly reduced. In nonexposed mice, vagal blockade significantly reduced all HRV parameters, suggesting that HRV is, in part, under vagal regulation in mice. Iron-soot exposure had no significant effect on resting membrane potential but decreased spiking responses of the identified cardiac vagal neurons to depolarizations (*p* < 0.05). The decreased spiking response was accompanied with a higher minimal depolarizing current required to evoke spikes and a lower peak discharge frequency.

**Conclusions:**

The data suggest that PM-induced neuroplasticity of cardiac vagal neurons may be one mechanism contributing to the cardiovascular consequences associated with PM_2.5_ exposure seen in humans.

The health effects of exposure to particulate matter (PM) have been well documented because of some major severe air pollution episodes (Bell and Davis 2001; Pope and Dockery 2006). Recently, PM-exposure–related cardiovascular effects have gained more attention. Epidemiologic studies show that, with less severe air pollution episodes, the association between ambient PM and cardiovascular deaths is stronger than that of PM and respiratory deaths (Brook et al. 2004; Pope and Dockery 2006). Fine PM [aero dynamic diameter ≤ 2.5 μm (PM_2.5_)] has been suggested to be the primary agent responsible for the deaths (Brook et al. 2003).

The cardiovascular causes of death, heart failure, arrhythmia, and ischemic heart disease have shown the strongest association with exposure to PM_2.5_ (Brook et al. 2004). The mechanism(s) underlying the PM_2.5_-exposure–induced adverse cardiovascular effects is not well understood. However, reduced autonomic function, which manifests as reduced heart rate variability (HRV), has emerged as a compelling potential cause (Pope and Dockery 2006). Even less understood is the cellular/biological mechanism(s) mediating PM-exposure–induced reduced HRV. Exposure to PM has been shown to alter central nervous system (CNS) signal processing. Perinatal environmental tobacco smoke (ETS) exposure increases the level of serotonin in the caudate nucleus (Slotkin et al. 2006) and changes receptor-mediated adenylyl cyclase signaling in the CNS (Slotkin et al. 2001). These data suggest that modulation of CNS activity may lead to impaired HRV.

Resolving the mechanisms of reduced HRV rests on understanding the regulation of HRV. HRV is dually regulated by the cardio-inhibitory vagal and the cardioexcitatory sympathetic branches of the autonomic nervous system. In the CNS, the cardiac vagal neurons are largely located in the nucleus ambiguus (NA) in the ventrolateral medulla (Corbett et al. 1999). Thus, these neurons could be a principle target for PM-induced modification of HRV.

Iron-soot is an ideal model for studying PM_2.5_ exposure. Soot and iron are ubiquitous small particulates found in the environment and can be reproducibly regulated from exposure to exposure. Transition metals, including iron, have been shown to be key mediators of PM-exposure–induced oxidative stress and lung inflammation (Costa and Dreher 1997; Zhou et al. 2003). The objectives of the present study were, first, to determine whether the inclusion of iron in PM has a greater effect on PM-exposure–induced decrease in HRV and, second, to test the hypothesis that PM_2.5_ impairs vagal regulation of HRV by decreasing the excitability of the cardiac vagal neurons in the nuclues ambiguus (NA).

## Materials and Methods

All protocols were approved by the Institutional Animal Care and Use Committee in compliance with the Animal Welfare Act (Office of Laboratory Animal Welfare 2002) and Public Health Service Policy on Humane Care and Use of Laboratory Animals (Animal Welfare Act 1966). All animals were treated humanely and with regard for the alleviation of suffering.

### Electrocardiogram (ECG) telemetry implants

Male C57BL/6 mice (10 weeks of age; Charles River Laboratories, Inc. Wilmington, MA) were anesthetized with intramuscular ketamine (90 mg/kg) and xylazine (12.5 mg/kg). An electrocardiogram (ECG) telemetry device (TA10EA F-20; Data Sciences International, St. Paul, MN) was implanted in the peritoneal cavity. The two ECG leads were tunneled subcutaneously. The negative lead of the transmitter was sutured to the upper right pectoris muscle near the shoulder, and the positive lead was sutured to the left lateral side of the xiphoid process. Animals were given carprofen (5 mg/kg, subcutaneously) for pain control.

### PM exposure

A diffusion flame system was used to generate an aerosol of soot and iron oxide (Yang et al. 2001). Iron was introduced by passing ethylene over liquid iron pentacarbonyl. Samples of the particles were collected on Teflon filters and 200-mesh holey carbon-coated copper grids. The particle size distribution was analyzed with a differential mobility analyzer. The mass concentration of iron particles was measured with X-ray fluorescence. The mice were randomly assigned to filtered air (FA) exposure (*n* = 16), soot-only exposure (*n* = 16; total suspended particles, 218 ± 9 μg/m^3^, 0% iron), or iron-soot exposure (*n* = 15; total suspended particles, 211 ± 4 μg/m^3^, 17% ± 1% iron).

### ECG recording protocols

**Figure 1:**
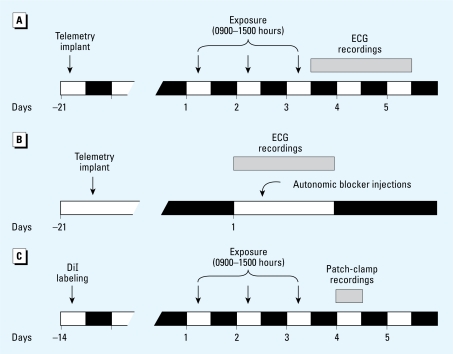
Experimental protocols. (*A*) Reduced HRV induced by PM_2.5_ exposure. (*B*) Effects of autonomic blockers on HRV. (*C*) Reduced excitability of cardiac vagal neurons induced by PM_2.5_ exposure.

As illustrated in Figure 1A, mice were exposed to FA or PM_2.5_ for 3 days (6 hr/day, 0900–1500 hours) 3 weeks after ECG telemetry device implant. Continuous ECG signals were recorded in freely moving mice for 48 hr after the last day of exposure (0800–1800 hours). The mice remained in their home cage throughout the exposure and recording period.

### Autonomic blockade on HRV

To determine the contribution of sympathetic and vagal regulation of HRV, we used a second group of mice without exposure. Continuous ECG signals were recorded before and after intraperitoneal injection (8 mL/kg) of saline (*n* = 8), 5% dimethyl sulfoxide (DMSO) solution (*n* = 7), sympathetic blocker (atenolol, 5 mg/kg; *n*= 8), vagal blocker (methylatropine, 2 mg/kg; *n* = 9), or a combination of atenolol and methylatropine (*n* = 8). Methylatropine was dissolved in DMSO and diluted to final concentration with normal saline. This protocol is illustrated in Figure 1B.

### *In vitro* electrophysiology

In a separate group of mice that did not have ECG telemetry implants, we tested PM-exposure–induced changes in neuronal behavior of the cardiac vagal neurons in the NA after 3 days of FA (*n* = 7 mice) or iron-soot (*n* = 8 mice) exposure (Figure 1C). The cardiac vagal neurons were retrogradely labeled with the fluorescent dye 1,1′-dioctadecyl 3,3,3′,3′ tetramethylindo-carbocyanine perchlorate (DiI) (Bouairi et al. 2006). Mice were anesthetized with ketamine (50 mg/kg) and xylazine (8 mg/kg). The heart was exposed via a left thoracotomy. A Parafilm patch coated with DiI was placed over the sinoatrial node and sealed with tissue glue. Animals were given carprofen (5 mg/kg, subcutaneously) for pain control. Mice were allowed to recover for 2 weeks before the exposure protocols.

The mice were anesthetized with ketamine (50 mg/kg) and xylazine (8 mg/kg) and decapitated. The brain was rapidly exposed and submerged in ice-cold high-sucrose artificial cerebrospinal fluid (aCSF) that contained (millimolar) 3 KCl, 2 MgCl_2_, 1.25 NaH_2_PO_4_, 26 NaHCO_3_, 10 glucose, 220 sucrose, and 2 CaCl_2_. Brainstem transverse slices (125 μm) were cut with a Leica VT1000 S vibrating microtome (Leica Microsystems, Inc. Bannockburn, IL). After incubation for 45 min at 37°C in high-sucrose aCSF, the slices were placed in normal aCSF that contained (millimolar) 125 NaCl, 2.5 KCl, 1 MgCl_2_, 1.25 NaH_2_PO_4_, 25 NaHCO_3_, 25 glucose, and 2 CaCl_2_. All experiments were performed at 33–34°C.

All whole-cell patch-clamp recordings were performed on fluorescently labeled cardiac vagal neurons in the NA. The neurons were visualized with infrared differential interference contrast (IR-DIC), and the fluorescence signal was visualized with an optical filter set for DiI (XF 108; Omega Optical Inc., Brattleboro, VT). Borosilicate glass electrodes were filled with a K-gluconate solution (millimolar) containing 130 K-gluconate, 1 NaCl, 1 MgCl_2_, 2 K-ATP, 0.3 Na-GTP, 1 CaCl_2_, 10 EGTA, and 10 HEPES. Recordings were made with a MultiClamp 700B amplifier (Axon Instruments, Sunnyvale, CA). Signals were filtered at 2 kHz and digitized at 10 kHz with the DigiData 1300A interface (Axon Instruments).

The cell was current-clamped at − 60 mV. Steady-state input resistance was determined with hyperpolarizing currents (100–400 pA, 200 msec). Neuronal spiking response was tested by injecting brief (1 sec) depolarizing current pulses (100–400 pA) and measuring total number of spikes evoked, the minimal current required to evoke spike, and the maximum peak frequency of the evoked spikes.

### Data acquisition and analysis

All values are means ± SE unless otherwise indicated. Differences were considered significant at *p* < 0.05. The ECG signals were recorded at 5 kHz with Dataquest A.R.T. (Data Sciences International). The raw data were converted to binary format with MiniAnalysis (Synaptosoft, Decatur, GA) and analyzed with Nevrokard SA-HRV software (Nevrokard Kiauta, Izola, Slovenia). The accuracy of the R-wave detection was visually confirmed. Only normal-to-normal RR intervals were used for HRV analysis in the time domain (Chen et al. 2008). The standard HRV parameters determined are listed in Appendix 1. In general, short-term HRV rMSSD (root mean square of successive difference) reflects alterations in autonomic tone that are predominantly vagally mediated (Kleiger et al. 1995). SDANN (standard deviation of all 2-min RR interval averages) reflects changes in both sympathetic and parasympathetic tone and provides information about the variability over a longer cycle, such as diurnal changes. SDNNIDX (averages of standard deviation of all 2-min RR intervals), considered an “intermediate” measure, reflects changes in HRV regulation for up to 2 min. SDNN (standard deviation of all normal-to-normal RR intervals) and CV% [coefficient of variance, 100 × (SDNN/mean RR)] reflect the variability due to a combination of long-, intermediate-, and short-term components.

The data were also divided into 12-hr sections for determining the exposure effects during light versus dark periods. We used a two-way repeated analysis of variance (ANOVA) to analyze the difference between FA- and PM_2.5_-exposed mice, and Fisher’s least-significant-difference test for pairwise comparison. The measures of HRV after 3 days of exposure are expressed as percent change from the averages of the FA control group. The measures of HRV during autonomic blockade are expressed as a percentage of the baseline before injection and were compared with a one-way ANOVA.

For *in vitro* electrophysiologic studies, an unpaired *t*-test was used to compare the resting membrane potential, input resistance, and minimal current required to evoke spikes. A two-way repeated ANOVA was used to determine the total number of spikes and the peak frequency. The peak frequency at the injected current that evoked 30–40 spikes was also determined and compared with an unpaired *t*-test.

## Results

Table 1 shows the 24-hr and the 12-hr light- and dark-period heart rate and HRV parameters recorded just after 3 days of FA exposure. The mice displayed a typical circadian rhythm having a higher RR interval and a higher HRV (all HRV parameters) during light period when the vagal regulation is expected to be higher.

### PM_2.5_ exposure reduced HRV

**Figure 2:**
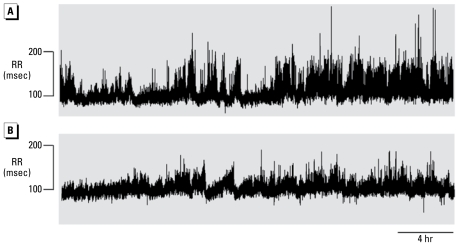
Example tachograms from individual mice after 3 days of exposure to FA (*A*) or PM in the form of iron-soot (*B*).

Exposure to PM_2.5_ for 3 days reduced measures of HRV in C57BL/6 mice. A representative tachogram from an FA-exposed control mouse and an iron-soot–exposed mouse recorded during the first 24 hr after 3 days of exposure are shown in Figure 2. The PM_2.5_-exposed mouse (Figure 2B) showed reduced HRV, as indicated by less frequent and lower magnitude “fluctuations” in the RR intervals.

**Figure 3:**
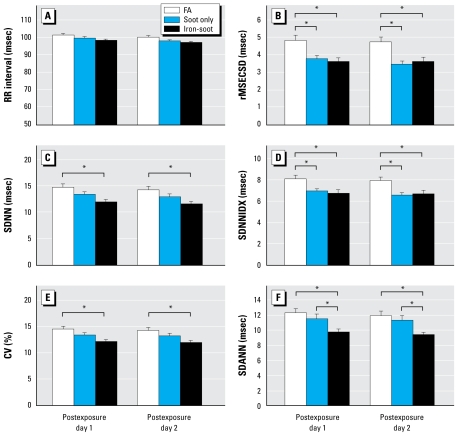
Group data (mean ± SE) of RR interval and 24-hr HRV in mice exposed to FA (*n* = 16), soot only (*n*= 16), and iron-soot (*n* = 15). *p < 0.05 between the two groups.

Three days of exposure to the PM_2.5_ in the form of soot only had no significant effect on the RR interval (Figure 3A) or SDNN (Figure 3B,C). However, rMSSD (Figure 3D) and SDNNIDX (Figure 3E) were significantly decreased on both days 1 and 2 of the postexposure period. In contrast, soot-only exposure had no significant effect on SDANN (Figure 3F). In the presence of iron in the PM_2.5_, there was a small (< 5%) but non- statistically significant increase in the RR interval (Figure 3A). Exposure to iron-soot significantly reduced all measures of HRV on both postexposure days 1 and 2 (Figure 3B–F) compared with FA control. In addition, SDANN was significantly lower in the iron-soot–exposed group than in the soot-only–exposed group (Figure 3F).

**Figure 4:**
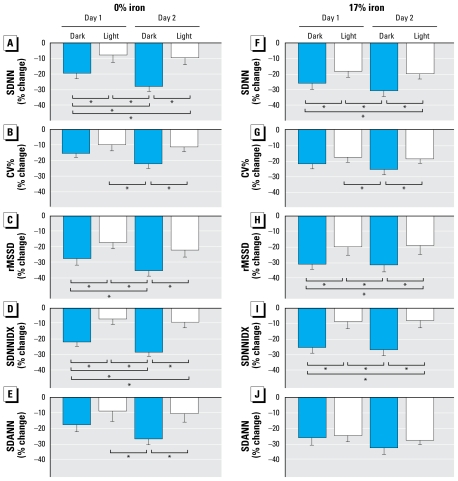
Group data of 12-hr HRV in mice exposed to PM_2.5_ with (*F–J*) or without iron (*A–E*). All data are expressed as percent changes from the average values of the FA-exposed control group (mean ± SE). *p < 0.05 between the two time points.

When effects were partitioned into dark and light postexposure periods, there was an overall soot-exposure–induced decrease in all measures of HRV (Figure 4A–E). The effects of PM_2.5_ exposure in the form of soot only were globally more prominent during the dark periods than during the light periods (Figure 4A–E). In addition, the decrease in HRV during the dark periods was greater on post exposure day 2 for overall HRV (Figure 4A), short-term HRV (Figure 4C), and HRV due to 2-min cycle length (Figure 4D), suggesting a lag time for the full effect of PM_2.5_ exposure.

The inclusion of iron also resulted in an overall exposure effect on all measures of HRV (Figure 4F–J). Similar to the effect of soot-only exposure, the exposure effect was more prominent during the dark periods (Figure 4F–I). In contrast to soot exposure, there was no obvious lag time in the PM-induced decrease in HRV, as shown by the absence of significant difference between effects on postexposure days 1 and 2 (Figure 4F–J).

### Effects of autonomic blockers on HRV

**Figure 5:**
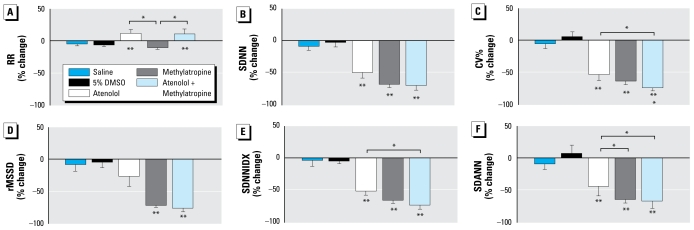
Group data of autonomic blockade on RR interval and HRV. All data are expressed as percent change from the baseline before the injection (mean ± SE). *p < 0.05 between the two groups. **p < 0.05 versus saline and 5% DMSO.

As expected, sympathetic blockade with atenolol significantly increased the RR interval, whereas parasympathetic blockade with methylatropine resulted in a nonsignificant decrease in the RR interval (Figure 5A). Blocking both sympathetic and parasympathetic limbs also increased the RR interval, suggesting that the baseline heart rate is chiefly under sympathetic influence (Figure 5A). Sympathetic blockade significantly decreased SDNN (Figure 5B,C), SDNNIDX (Figure 5E), and SDANN (Figure 5F) but had no significant effect on rMSSD (Figure 5D). On the other hand, parasympathetic blockade decreased all measures of HRV (Figure 5B–F). The combined blockade also significantly reduced all measures of HRV (Figure 5B–F). Both vehicle controls had no consistent effect on heart rate and measures of HRV. The data suggest that HRV is under both cardiac sympathetic and vagal regulation, with a greater influence from cardiac vagal inputs.

### PM_2.5_ exposure reduced excitability of cardiac vagal neurons

**Figure 6:**
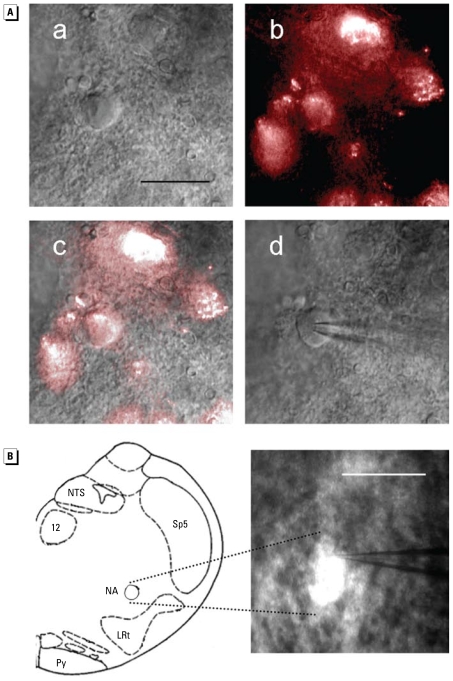
(*A*) An identified cardiac vagal neuron viewed at 40×: the neuron viewed with IR-DIC (a), the neuron viewed with fluorescence filter set (b), overlay of the IR-DIC and fluorescence images (c), and neuron with patch electrode in whole-cell configuration (d). Bar = 50 μm. (*B*) Schematic drawing showing composite of recording sites (left) and the brainstem slice viewed at 5×. Abbreviations: LRt, lateral reticular nucleus; NA, nucleus ambiguus; NTS, nucleus tractus solitarii; Py, pyramidal tract; Sp5, spinal trigeminal nucleus; 12, hypoglossal nucleus. Bar = 500 μm.

**Figure 7:**
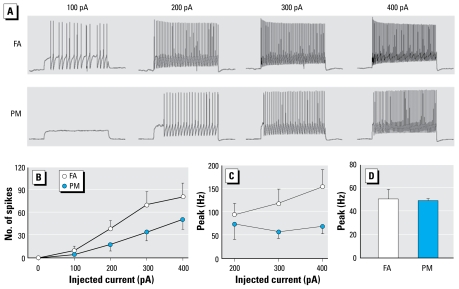
Neuronal response to intracellular depolarizing current (100–400 pA) injections in FA-exposed control and PM-exposed mice. (*A*) Examples of the spiking response to depolarizing current injections. (*B*) Group data (mean + SE) showing the depolarizing current evoked total number of spikes in FA- and PM-exposed mice. (*C*) Group data (mean + SE) of instantaneous peak frequency at each depolarizing current. (*D*) Group data (mean + SE) of instantaneous peak frequency at the depolarizing current that evokes 30–40 spikes.

Our data suggest that the cardiac vagal limb of the autonomic nervous system plays an important role in HRV regulation and that the PM_2.5_-exposure–induced decrease in HRV may be due to exposure-induced neuroplasticity in cardiac vagal neurons. To better define the cellular mechanisms underlying the PM_2.5_-exposure–induced decrease in HRV, we performed electrophysiologic experiments on cardiac vagal neurons in the NA that were identified by the presence of fluorescence dye (Figure 6). PM_2.5_ exposure in the form of iron-soot decreased neuronal excitability to depolarizing current injections (100–400 pA). Figure 7A shows examples of the spiking responses of cardiac vagal neurons from an FA and a PM-exposed mouse. The neuron from the PM-exposed mouse discharged fewer spikes than did the FA-exposed mouse at the same injected currents. The group data (Figure 7B) illustrate that the total number of spikes discharged in response to depolarizing current injections was significantly lower in the mice exposed to PM_2.5_ (two-way ANOVA: exposure, *p* = 0.071; current, *p* < 0.001; interaction, *p* = 0.018).

The decrease in spiking response was accompanied with a higher minimal depolarizing current required to evoke spikes (FA, 155 ± 21 pA; PM, 223 ± 23 pA; *p* = 0.041). There was a small nonsignificant increase in action potential threshold (FA, − 36 ± 3 mV; PM, − 32 ± 2 mV; *p* = 0.199). The instantaneous peak frequency of the spike discharge (two-way ANOVA: exposure, *p* = 0.073; current, *p* < 0.001; interaction, *p* = 0.009) was lower in PM-exposed mice (Figure 7C). However, there was no difference in the instantaneous peak frequency between the two groups at the depolarizing current that evokes 30–40 spikes (*p* = 0.8221; Figure 7D).

PM_2.5_ exposure had no significant effect on the resting membrane potential (FA, − 53 ± 3 mV; PM, − 54 ± 2 mV; *p* = 0.706), input resistant (FA, 154 ± 17 MΩ; PM, 149 ± 19 MΩ; *p* = 0.183), or cell capacitance (FA, 47 ± 6 pF; PM, 51 ± 3 pF; *p* = 0.630).

## Discussion

The major findings of the present study are that a short-term (3-day) exposure to PM_2.5_ in the form of iron-soot or soot only results in a significant reduction in HRV that persists at least 48 hr after the exposure ceases. PM_2.5_ exposure also significantly decreased the spiking responses and peak discharge frequency of cardiac vagal neurons. These data suggest that PM-induced neuroplasticity of cardiac vagal neurons may be one mechanism contributing, via reduced HRV regulation, to the cardiovascular consequences associated with PM_2.5_ exposure. The exposure-induced reduced HRV is greater with the inclusion of iron, one of the transition metals that have been shown to be key mediators of PM-exposure–induced oxidative stress and lung inflammation (Costa and Dreher 1997; Zhou et al. 2003). These data suggest that transition metals may exaggerate the reaction in the lung to enhance PM-exposure–induced cardio vascular consequence.

### HRV regulation

Epidemiologic studies consistently illustrate a significant association between PM exposure and HRV (Pope and Dockery 2006). Here, we confirm in mice that, as is the case in humans, short-term HRV is mostly under cardiac parasympathetic regulation, whereas the sympathetic limb has greater influence on the baseline heart rate. Blocking the cardiac parasympathetic modu lation has no effect on heart rate but significantly decreases all HRV parameters, as previously reported (Gehrmann et al. 2000). Blockade of cardiac sympathetic modulation significantly decreased heart rate as well as some HRV parameters while having no significant effect on short-term HRV. HRV analysis in frequency domain has been demonstrated in humans to be a powerful tool for isolating the contribution of sympathetic versus parasympathetic control of HRV. Unlike the HRV analysis in time domain, stationarity is the key for frequency domain analysis. In the present study, challenges in analyzing HRV in frequency domain could have jeopardized the interpretation of the frequency-domain data. First, we conducted the recordings over a long period of time, which presents the unavoidable issue of nonstationarity. Selecting short periods of recordings for frequency analysis is not desirable because this could introduce bias/error through data selection. Second, the mice in the present study were freely moving in their home cage, where changes in breathing rate could occur from moment to moment. Changes in breathing rate could have significant effects on high-frequency power. Given these considerations, we chose to perform only time domain analysis.

### Reduced neuronal excitability on HRV regulation

Although the exposure-induced decrease in short-term HRV suggests a reduced vagal regulation of the heart, the CNS cellular mechanism(s) underlying the exposure-induced reduction in HRV remains unclear. The nucleus tractus solitarii (NTS) is the first central site that integrates information from the cardiovascular system. We have previously shown that exposure to allergen and/or ozone can induce central neuronal plasticity in the NTS (Chen et al. 2001, 2003). We have further shown that chronic ETS exposure alters the synaptic transmission in the NTS (Sekizawa et al. 2008). In the present study, we demonstrate that PM_2.5_ exposure decreases excitability of the cardiac vagal preganglionic neurons in the NA. These data suggest that PM_2.5_ exposure reduces HRV, in part, by changing neuronal behavior of the final output from the CNS.

The decreased spiking response to depolarization suggests that these cardiac vagal neurons have a muted responsiveness to inputs from upstream regions, such as the NTS. As the magnitude of the depolarization was increased, the blunting effects of PM_2.5_ exposure became more prominent. The data suggest that acute robust volleys of activation will evoke a disproportionately smaller output. Given that these cardiac vagal neurons regulate cardiac parasympathetic efferent nerve activity, and given that the sympathetic and parasympathetic limbs dually regulate HRV, the decreased cardiac parasympathetic output may allow a greater influence of the sympathetic regulation of the heart. The influence of same sympathetic activity on heart rate is greater when the parasympathetic activity is lower (Levy and Zieske 1969). Therefore, PM exposure could reduce HRV directly by reducing parasympathetic regulation and indirectly by exaggerating the sympathetic influence.

### Potential mechanisms inducing central neuroplasticity

It is likely that multiple mechanisms contribute to PM_2.5_-exposure–induced cardiovascular-related morbidity and mortality. Pulmonary and/or systemic inflammatory responses, enhanced coagulation/thrombosis, vascular endothelial dysfunction, atherosclerosis, cardiac malfunction, and autonomic dysfunction have been suggested (Brook et al. 2004; Pope and Dockery 2006). In terms of the neuroplasticity seen in the present study, inhaled PM may influence neuronal behavior by various physiological or biochemical pathways. Inflammatory mediators may increase the excitability of lung sensory nerves that synapse, directly or indirectly, onto these cardiac vagal neurons (Undem et al. 1993). Such changes in synaptic traffic are known to induce plasticity in CNS neurons (Debanne et al. 2003). Circulatory inflammatory mediators may access CNS neurons devoid of a blood–brain barrier that send projections to the NA (Morest 1967). In addition, inhaled PM and/or the inflammatory mediators may disrupt the blood–brain barrier to gain direct access to these cardiac vagal neurons (Calderon-Garciduenas et al. 2002).

## Conclusion

Exposure to PM_2.5_ reduced HRV, and the inclusion of iron enhanced the exposure effects on HRV. The present study demonstrates, for the first time, that PM_2.5_ exposure reduces neuronal responsiveness to excitation in anatomically identified cardiac vagal neurons in the NA. The data suggest that PM-induced decreases in cardiac vagal neuronal excitability may be one mechanism contributing to the cardiovascular consequences associated with PM_2.5_ exposure seen in humans.

## Figures and Tables

**Table 1:** Heart rate and HRV in FA control mice (*n* = 16).

Measure	24 hr	12-hr light period	12-hr dark period
RR (msec)	100.7 ± 3.8	104.2 ± 4.2	97.5 ± 4.5*
SDNN (msec)	14.6 ± 2.5	15.4 ± 2.9	12.8 ± 2.0*
CV%	14.5 ± 2.2	14.7 ± 2.3	13.0 ± 1.7*
rMSSD (msec)	4.8 ± 1.1	5.2 ± 1.4	4.3 ± 0.9*
SDNNIDX (msec)	8.1 ± 1.4	9.1 ± 1.9	7.2 ± 1.0*
SDANN (msec)	12.2 ± 2.4	12.1 ± 2.5	10.7 ± 2.0*

* *p* < 0.05, light cycle versus dark period, paired *t*-test.
